# Genetic and functional homologous repair deficiency as biomarkers for platinum sensitivity in TNBC: A case report

**DOI:** 10.3389/fonc.2022.963728

**Published:** 2022-09-14

**Authors:** Diego Gomez-Puerto, Alba Llop-Guevara, Mara Cruellas, Sara Torres-Esquius, Javier De La Torre, Vicente Peg, Judith Balmaña, Isabel Pimentel

**Affiliations:** ^1^ Medical Oncology Department, Vall d’Hebron Institute of Oncology (VHIO), Hospital Universitari Vall d’Hebron, Vall d’Hebron Barcelona Hospital Campus, Barcelona, Spain; ^2^ Experimental Therapeutics Group, Vall d’Hebron Institute of Oncology (VHIO), Vall d’Hebron Barcelona Hospital Campus, Barcelona, Spain; ^3^ Hereditary Cancer Genetics Group, Vall d’Hebron Institute of Oncology (VHIO), Hospital Universitari Vall d’Hebron, Vall d’Hebron Barcelona Hospital Campus, Barcelona, Spain; ^4^ Gynecologic Oncology and the Breast Pathology Unit, Hospital Universitari Vall d’Hebron, Vall d’Hebron Barcelona Hospital Campus, Barcelona, Spain; ^5^ Pathology Department, Vall d’Hebron University Hospital, Barcelona, Spain; ^6^ Breast Cancer and Melanoma Group, Vall d’Hebron Institute of Oncology (VHIO), Hospital Universitari Vall d’Hebron, Vall d’Hebron Barcelona Hospital Campus, Barcelona, Spain

**Keywords:** RAD51, triple-negative breast cancer, HRD-biomarkers, RAD51D, pathological complete response (pCR)

## Abstract

Triple-negative breast cancer is the most aggressive subtype of mammary carcinoma. In the early stage, neoadjuvant chemotherapy (NAC) is the standard of care for prognostic stratification and the best adjuvant treatment strategy. A 30-year-old female presented in the emergency room because of a gigantic right breast associated with an ulcerated lump at the upper quadrants. The right axillary nodes were palpable. An ultrasound was performed, showing the ulcerated neoformation with enlarged right axillary lymph nodes observed to level III. A core biopsy of the breast lesion was performed, and the pathological examination revealed a nonspecial type, grade 3, invasive, triple-negative breast cancer. No distant disease was found in the PET-CT scan. A germline genetic panel by next-generation sequencing identified a likely pathogenic variant in *RAD51D* (c.898C>T). Assessment of the functionality of the DNA homologous recombination repair pathway by RAD51 foci in the tumor revealed a profile of homologous recombination deficiency. NAC consisting of weekly carboplatin and paclitaxel followed by dose-dense doxorubicin/cyclophosphamide was performed with a complete metabolic response achieved in the PET-CT scan. The patient underwent a modified radical mastectomy plus axillary lymphadenectomy with a pathological complete response in the breast and axilla and remains disease-free after 2 years of follow-up. We report a young female with a triple-negative breast cancer stage cT4bN3M0 and a hereditary pathogenic mutation in *RAD51D*. The tumor was highly proliferative and homologous recombination-deficient by *RAD51*. The patient received platinum-based NAC, achieving a pathologic complete response. More effort should be made to identify predictive functional biomarkers of treatment response, such as RAD51 foci, for platinum sensitivity.

## Introduction

Triple-negative breast cancer (TNBC) is defined by the lack of expression of the estrogen receptor (ER), progesterone receptor (PR), and human epidermal growth factor receptor 2 (HER2) by immunohistochemistry. It is commonly associated with an aggressive clinical course and worse outcomes when compared with other subtypes of breast cancer. TNBC represents 10%–15% of all breast cancers and occurs in younger women, usually under 40 years of age (1). Poor prognosis is mainly seen in patients who do not achieve a pathologic complete response (pCR) after neoadjuvant treatment with a risk of relapse between 40% and 50% if pCR is not achieved (2, 3).

Up to 15%–20% of patients with TNBC harbor a breast cancer susceptibility gene (*BRCA*) mutation, particularly in *BRCA1 or BRCA2* (4). Nevertheless, other encoding pathogenic gene variants and epigenetic silencing of genes related to the DNA homologous recombination repair (HRR) pathway have been described, including *PALB2*, *CHEK2*, *RAD51C*, and *RAD51D* (5).

Tumor HRR deficiency (HRD) leads to irreparable DNA damage from platinum-containing agents, which leads to cell death and marks an underlying cell sensitivity to polyadenosine diphosphate–ribose polymerase inhibitors (PARPi) (6). The assessment of HRD in the clinic measures a genotype and can currently be divided into three testing method categories: HRR gene–based tests, genomic scars and signatures, and functional assays (7). HRR gene–based tests attempt to detect germline or homozygous somatic mutations in *BRCA1* or *BRCA2* and *non-BRCA* HRR genes as mentioned before. Measuring the different genomic signatures and scar features produced by tumoral cells and displayed in abnormal copy number profiles and thousands of somatic mutations genome-wide, characterized principally by short deletions and short tandem duplications, provides ways of identifying cancers with a history of HRD irrespective of the underlying etiology. Functional assays have the potential to provide a dynamic readout of actual, extant, HRR status (7). The most commonly used experimental system to estimate HRR is to estimate the amount of nuclear RAD51 foci as was done in our patient.

Preclinical studies support the determination of RAD51 nuclear foci as a functional and dynamic biomarker of HRD to identify *BRCA*-mutated and other HRR-altered breast cancer genes. This functional HRD, defined as a RAD51 low score (≤10% RAD51+/GMN+ cells), can also predict a benefit from DNA damaging agents, such as platinum-based chemotherapy and PARPi (8, 9).

More recently, *RAD51* was able to identify TNBC patients from the GeparSixto trial who benefited from adding carboplatin in the neoadjuvant chemotherapy (NAC) setting (10). In particular, the pCR rate in RAD51 low tumors was 33% after treatment with paclitaxel, doxorubicin, and bevacizumab, whereas it was 66% when adding carboplatin (OR 3.96, 1.56–10.05, *p* = .004). Functional HRD by *RAD51* was identified in 93% of *BRCA*-mutated tumors and 45% of non-*BRCA* mutant cases, providing evidence of HRD beyond *BRCA* mutation and in a large proportion of TNBC tumors (10).

In this case report, we describe a very high-risk patient presenting with an enormous and ulcerated TNBC with both a functional HRD by *RAD51* as well as a germline *RAD51D* PV with pCR to platinum-based NAC.

## Case presentation

A 30-year-old woman presented to the emergency room after noticing an enlarged right breast associated with a mass for the past 6 months with progressive growth. The patient had been in her usual health before this presentation.

**Figure 1:**
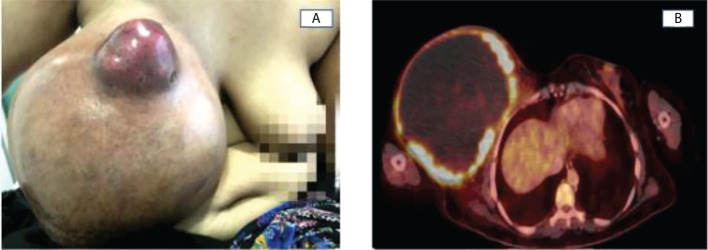
**(A)** Enlarging right breast cancer with a painless, ulcerated, hard, irregular mass in the union of the upper quadrants. **(B)** Voluminous hypermetabolic mass of 18.4x15.6cm, with a cystic component in the right breast and a SUVmax >4.

She had no relevant past medical history and no family history of breast or ovarian cancer. Physical examination showed a gigantic right breast, more than four times larger than its counterpart associated with an ulcerated, hard, and erythematous nodule of 6 x 6 cm within the union of the upper quadrants (**Figure 1A**). At least three right axillary nodes were palpable and painless. Left breast exploration and the remainder of the physical examination were unremarkable. Blood test analyses revealed normocytic anemia and an elevated C-reactive protein.

**Figure 2:**
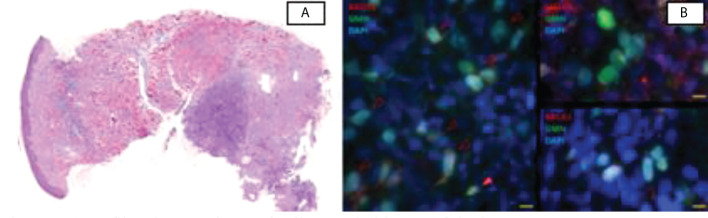
**(A)** Infiltrating carcinoma in diagnostic biopsy of the breast mass. **(B)** Representative images of the IF assay to detect biomarkers in the tumor. Geminin (GMN) and DAPI identify cells in S/G2 cell cycle phase. *Left*, RAD51 nuclear foci are only found in normal cells (closed triangle) but not in tumor cells (open triangles). *Right*, abundant dsDNA damage (γH2AX foci) and BRCA1 foci in tumor cells. *Scale*, 10 µm.

Ultrasonography was performed, confirming a right breast lesion with a very large cystic component. Adenopathy involvement at its three levels was perceived at the right axilla. In the left breast, no lesions or adenopathy involvement were seen. A core biopsy was performed of one of the right axillary nodes and the breast lesion. Pathological examination of the specimen exposed no special type histologic grade 3 invasive triple-negative breast cancer in both biopsied tissues with a Ki67 of 75%. Stromal tumor-infiltrating lymphocytes were low (5%) (**Figure 2A**).

PET-CT showed a voluminous hypermetabolic mass in the right breast, compatible with a primary tumor, with locoregional lymph node involvement and without any distant lesions (**Figure 1B**). Tumoral biomarkers were done with a CEA of 4 ng/ml and an elevated CA 15-3 of 436 U/ml.

NAC with weekly carboplatin AUC2 and paclitaxel 80 mg/m^2^ for 12 weeks, followed by biweekly dose-dense Adriamycin (Doxorubicin) 60 mg/m^2^ IV and Cyclophosphamide 600 mg/m2 IV (ACdd) for four cycles was indicated.

Germline next-generation sequencing (NGS) breast cancer predisposition panels have enabled the detection of a wide spectrum of mutations and the characterization of the genomic profile. A hereditary OncoKitDx gene panel (Imegen) on a MiSeq (Illumina) was performed, which detects point mutations, small and large insertions, and deletions along 50 selected genes, identifying a likely PV (c.898C>T) in the *RAD51D* gene in heterozygosis. Furthermore, the *RAD51* test was performed as part of a unit research protocol, including all TNBC undergoing NAC. *RAD51* foci were assessed by immunofluorescence on a formalin-fixed paraffin-embedded breast tissue biopsy that was laid on a tissue microarray format. BRCA1 foci (as a mediator of HRR) and gH2AX foci (as a biomarker of endogenous double-strand DNA damage) analysis was also performed with each biomarker counterstained with geminin (as a marker of the S/G2 cell cycle phase) and 40, 6-diamidino2-phenylindole (DAPI). Manual scoring on an epifluorescence microscope resulted in a low RAD51 score (1% of GMN+ cells with ≥5 RAD51 foci), indicating HRD (**Figure 2**). High levels of yH2AX (50%57%) and BRCA1 (90%93%) were found, consistent with the same phenotype.

**Figure 3:**
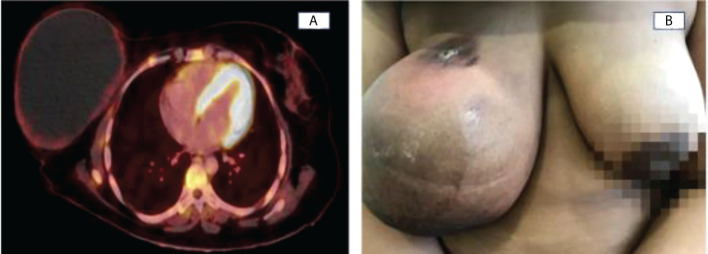
**(A)** Complete metabolic response due to persistence of a voluminous cystic left breast lesion with a thin wall of low uptake of FDG ((SUVmax:2,21) and complete disappearance of ipsilateral locoregional lymphadenopathy. **(B)** After completing NAC, the nodule at the union of the upper quadrants disappeared.

A rapid and progressive descent of CA15-3 was detected during the treatment, down to 146 U/ml after only six cycles of weekly carboplatin and paclitaxel, descending to 51 U/ml when finishing the 12 weeks of the weekly scheme. CA15-3 reached values within the normal range after two cycles of ACdd. Taking into account the extensive local disease, a conservative surgery was not indicated, for which chemotherapy efficacy was assessed by clinical examination before each cycle and by performing a PET-CT after concluding NAC. Taking into account the complexity of the tuoral mass and the high risk of local and distant disease progression, NAC efficacy was assessed by performing a PET-CT after concluding NAC, showing a complete metabolic response but the persistence of the voluminous cystic right breast lesion with complete disappearance of the ipsilateral locoregional lymphadenopathies (**Figures 3A, B**).

**Figure 4:**
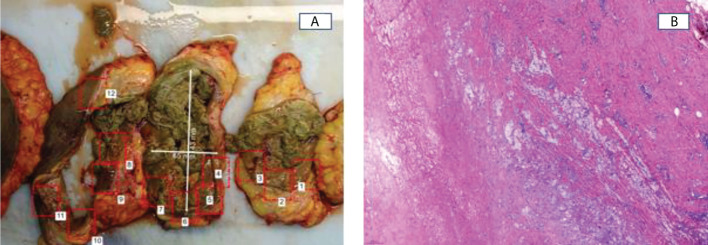
**(A)** Cystic cavity with necrohemorrhagic content covered by a thick fibrous wall with abundant sero-hemorrhagic fluid. **(B)** Histopathological examination of mastectomy specimens. No tumoral cells were found.

The patient underwent a modified radical mastectomy plus axillary lymphadenectomy (**Figure 4A**). Pathologic examination of the surgical specimen showed pCR in the breast and in the 14 lymph nodes removed. Secondary changes due to previous treatment were evident. These included a cystic cavity with necrohemorrhagic content lined by a dense fibrous wall with a moderate lymphohistiocytic component and hemosiderin deposits, secondary to old bleeding, without evidence of residual neoplastic cellularity (**Figure 4B**). ypT0N0 Residual Cancer Burden: 0. After surgery, the patient received radiotherapy with a total dose of 40.05 Gy in 15 sessions and remains with no evidence of disease 24 months after surgery.

## Discussion

TNBC is more likely to be diagnosed clinically rather than in screening mammograms because of its high proliferative index in a population that is under the age of usual screening assessments (11, 12).

A clinical presentation as a complex solid and cystic mass related to breast cancer is uncommon (13). Solid malignant masses can secrete mucin or fluid related to necrosis, which could result in complex, solid, and cystic morphology (14). Most histologies reported are NTS carcinoma, although papillary, mucinous, and adenoid cystic breast carcinomas are also associated without any predominance of the hormonal status described.

NAC adds a substantial prognostic value to patients with TNBC compared with those with ER positive disease with a larger reduction in the risk of recurrence. pCR is a known independent prognostic factor in TNBC (15, 16) with a disease-free survival (DFS) of 64 months in those achieving pCR (*p* <.001) compared with 42.4 months (*p* <.001) in the non-pCR group (17).

The regimen of NAC in TNBC, namely, the addition of platinum to the taxane-anthracycline–based chemotherapy is still a matter of debate. A meta-analysis including nine RCTs (*N* = 2109) demonstrated a significant 15.1% increased pCR rate with the use of platinum-based chemotherapy (OR 1.96, 95% CI 1.46–2.62, *P* <.001) (18, 19). The benefit of adding carboplatin was even more pronounced when restricted to patients who received the current standard NAC with weekly paclitaxel followed by anthracycline and cyclophosphamide (21.6% increased pCR rate; OR 2.53, 95% CI 1.37–4.66, *P* = .003) (19).

The rationale for platinum-based chemotherapy in TNBCs is related to the high quantity of genetic or epigenetic variations in the HRR pathway observed in these tumors that leads to a deficiency in double-stranded DNA repair. The alterations include germline *BRCA 1/2 *mutations; hypermethylation of the *BRCA1* promoter; and mutations in *PALB2*, *RAD51C*, and *RAD51D* or epigenetic silencing of *RAD51C* and *BRCA1*  (8). HRD impairs regular DNA damage repair, which results in loss or duplication of chromosomal regions generating a genomic loss of heterozygosity (LOH) (6).

Germline pathogenic variants in *RAD51C/D* with loss of tumor heterozygosity have been associated with a molecular phenotype compatible with deficient HRD by genomic signatures (HRD score or genomic LOH) (20).

Functional HRD is shown to correlate to the HRR mutations/*BRCA1* methylation status (9, 21–23). Moreover, RAD51 identifies more than 90% *of BRCA*-mutated tumors and 45% of non-*BRCA* mutant cases as functional HRD, opening a therapeutic window for both platinum agents and PARPi in a wider population. Detection of high RAD51 foci in germline *BRCA *tumors has been suggested as a biomarker of platinum and PARPi resistance regardless of the underlying mechanism restoring HRR function (9, 23). Furthermore, a functional HRD by RAD51-low potentially benefits more in terms of pCR (pCR 66% vs. 33%, OR 3.96, 1.56-10.05, p: 0.004) when using a platinum-based NAC (10). Further clinical trials and results are needed to support these findings and to clear the benefit in terms of DFS and OS.

In the case presented here, the patient had both a *RAD51D* PV (a nonsense variant predicted to result in an early protein truncation) and a RAD51-low score, which could justify the outstanding response to the NAC.

Nevertheless, since the KEYNOTE-522 trial results, pembrolizumab in addition to NAC yielded an absolute benefit of 13.6% in pCR and 7.7% in event-free survival (HR 0.63; 95% CI, 0.48 to 0.82; *p* <.001), becoming the new standard of care for unselected early-stage TNBC (24, 25). However, at the time this patient initiated chemotherapy, those results were pending to be presented. Interestingly, this patient achieved pCR, opening the research question of a potential de-escalation of treatment in selected *BRCA* mutant or RAD51-low score TNBC, with which an excellent response to platinum-based chemotherapy or other targeted therapies as PARPi is expected.

Several randomized trials are examining the benefit of the addition of carboplatin with or without PARPi to NAC in TNBC. The BrighTNess trial evaluated the addition of the PARP inhibitor veliparib plus platinum-based NAC showing that the proportion of patients who achieved a pCR in the paclitaxel, carboplatin, and veliparib group were not improved compared with patients receiving platinum-taxane-based NAC without veliparib (92 [58%] of 160 patients, *p* = .36) (26). In the Neotala trial presented at the 2021 ASCO annual meeting (27), germline BRCA-mutant stage I–III TNBC received neoadjuvant single-agent talazoparib for 24 weeks, achieving a 49.2% pCR of the intention-to-treat population, comparable to that observed with the combination of anthracycline and taxane-based chemotherapy. Additional results are needed to clarify a possible role of PARPi in the TNBC neoadjuvant setting.

Furthermore, the OlympiA trial has demonstrated a clear invasive disease-free survival (IDFS) benefit with 1 year of adjuvant olaparib (vs. placebo) in patients with germline *BRCA1/2* mutations with residual invasive disease after NAC (HR 0.58; 95% CI, 0.41–0.82; *p* <.001). The inquiry of a potential benefit of adjuvant PARPi in functional HRD despite normal *BRCA1/2* expression in high-risk patients remains unclear (28).

## Conclusions

TNBC affects young women and is characterized by its rapid growth and high risk of relapse if pCR is not achieved. The presence of HRD tumors beyond *BRCA* mutation creates a potential window of opportunity to escalate but also de-escalate treatment.

In this case report, germline *RAD51D* PV and RAD51 low scores likely determined the chemotherapy sensitivity observed. More research is needed on functional and dynamic biomarkers, such as the RAD51 test, to validate its predictive value on drug responsiveness.

## Data availability statement

The original contributions presented in the study are included in the article/Supplementary Material. Further inquiries can be directed to the corresponding author.

## Ethics statement

The studies involving human participants were reviewed and approved by the Clinical Research Ethics Committee of Vall d’Hebron Hospital. The patients/participants provided their written informed consent to participate in this study. Written informed consent was obtained from the individual(s) for the publication of any potentially identifiable images or data included in this article.

## Author contributions

DGP and IP conceived and developed the manuscript and provided **Figures 1A** and **3B**. DGP contributed **Figures 1B** and **3A**. ALP and STE contributed to performed the molecular analyses and interpretation of the results. ALP assessed RAD51 foci by immunofluorescence on the formalin-fixed paraffin-embedded breast tissue biopsy performing a manual scoring on an epifluorescence microscope and contributed **Figure 2B**. MC and JB did the clinical genetic evaluation and revised the manuscript. JT performed the initial clinical assessment and performed the surgery. VP did the pathological examination and contributed **Figures 2A** and **4B**. Germline next-generation sequencing (NGS) breast cancer predisposition panel was performed as part of routine care in our center in order to find germline mutations in patients with triple-negative breast cancer. The RAD51 test was performed as part of a unit research protocol including all TNBC undergoing neoadjuvant chemotherapy. All authors contributed to the article and approved the submitted version.

## Acknowledgments

The authors recognize and express gratitude to the patient and her family. The VHIO authors acknowledge the Cellex Foundation for providing research facilities and thank CERCA Programme/Generalitat de Catalunya for institutional support.

## Conflict of interest

The authors declare that the research was conducted in the absence of any commercial or financial relationships that could be construed as a potential conflict of interest.

## Publisher’s note

All claims expressed in this article are solely those of the authors and do not necessarily represent those of their affiliated organizations, or those of the publisher, the editors and the reviewers. Any product that may be evaluated in this article, or claim that may be made by its manufacturer, is not guaranteed or endorsed by the publisher.
